# Interaction of Mycobacteria With Host Cell Inflammasomes

**DOI:** 10.3389/fimmu.2022.791136

**Published:** 2022-02-14

**Authors:** Shivangi Rastogi, Volker Briken

**Affiliations:** Department of Cell Biology and Molecular Genetics, University of Maryland, College Park, MD, United States

**Keywords:** Mycobacterium tuberculosis, inflammasome, NLRP3, AIM2, ESX-1, IL-1b, NTM = nontuberculous mycobacteria

## Abstract

The inflammasome complex is important for host defense against intracellular bacterial infections. *Mycobacterium tuberculosis* (Mtb) is a facultative intracellular bacterium which is able to survive in infected macrophages. Here we discuss how the host cell inflammasomes sense Mtb and other related mycobacterial species. Furthermore, we describe the molecular mechanisms of NLRP3 inflammasome sensing of Mtb which involve the type VII secretion system ESX-1, cell surface lipids (TDM/TDB), secreted effector proteins (LpqH, PPE13, EST12, EsxA) and double-stranded RNA acting on the priming and/or activation steps of inflammasome activation. In contrast, Mtb also mediates inhibition of the NLRP3 inflammasome by limiting exposure of cell surface ligands *via* its hydrolase, Hip1, by inhibiting the host cell cathepsin G protease *via* the secreted Mtb effector Rv3364c and finally, by limiting intracellular triggers (K^+^ and Cl^-^ efflux and cytosolic reactive oxygen species production) *via* its serine/threonine kinase PknF. In addition, Mtb inhibits the AIM2 inflammasome activation *via* an unknown mechanism. Overall, there is good evidence for a tug-of-war between Mtb trying to limit inflammasome activation and the host cell trying to sense Mtb and activate the inflammasome. The detailed molecular mechanisms and the importance of inflammasome activation for virulence of Mtb or host susceptibility have not been fully investigated.

## Introduction

Tuberculosis (TB) is a major cause of morbidity and mortality with approximately 10 million new cases and 1-2 million deaths, annually (1). The disease is caused by the human pathogen *Mycobacterium tuberculosis* (Mtb) which is transmitted *via* aerosol from the lung of an infected individual to the naïve bystander. Current chemotherapy leads to positive outcomes in about 85% of the patients but takes 6-9 months to complete and the success rate drops dramatically if drug resistant strains of Mtb are the cause of the infection (2). Consequently, the search for better antibiotics and more efficient treatment regiments is of great interest. In addition, host-directed therapy (HDT) is a complementary approach to improving clinical outcomes by targeting host signaling pathways that, for example, support bacterial replication or cause immune pathologies (3). For the latter approach to be successful, a more detailed understanding of host responses to infection with Mtb and their importance for host protection or susceptibility is required. The cytokine Interleukin (IL)-1β is of crucial importance for host resistance to Mtb. In this review we will provide some background information on mechanisms of inflammasome activation which leads to the generation of IL-1β, summarize the findings of the importance of IL-1β for host resistance to Mtb infections and their potential for host-targeted therapeutic approaches and finally we will focus on how the inflammasome detects various mycobacterial species and how Mtb is able to inhibit inflammasome activation (summarized in **Table 1** and **Figure 2**).

**Table 1:** Summary overview of different mycobacterial species and effectors involved in activation of either NLRP3 or AIM2 inflammasome.

Pathogen	Strains	Inflammasome target	Cell Type/*in vivo*	Mechanism/Function/Triggers involved	Bacterial effector/mediators	References
*M. tuberculosis*	H37Rv	NLRP3	peritoneal exudate macrophages, BMDMs	Induction of Potassium efflux results in increased secretion of IL-1β and IL-18	RD1 Locus	(4)
*M. tuberculosis*	H37Rv	NLRP3	J774A.1, BMDMs, and THP-1 macrophages	Assembly of NLRP3 inflammasome complex (interacts with NATCH and LRR domains)	PPE13	(5)
*M. tuberculosis*	H37Rv	NLRP3	mouse retinal pigment epithelium (RPE) cells	Caspase-1 activation	EsxA and dsRNA	(6)
*M. tuberculosis*	H37Rv	NLRP3	PBMCs, THP-1 macrophages	Up-regulates expression of MFN2 and induces release of IL-1β	EsxA	(7)
*M. tuberculosis*	H37Rv ATCC 27294	NLRP3	Mouse peritoneal macrophages, PMA-differentiated THP1 cells, and BMDMs	Induces GSDMD mediated pyroptosis through interaction with RACK-1	Rv1579c (EST12)	(8)
*M. tuberculosis*	H37Rv	NLRP3	BMDMs	Phagocytosis and Potassium efflux	RD1 locus	(9)
*M. tuberculosis*	H37Rv ATCC 25618	NLRP3	THP-1 macrophages,BMDMs, BMDCs	induces Caspase-1 activation and IL-1β secretion	ESX-5a	(10)
*M. tuberculosis*	H37Rv	NLRP3	THP-1 macrophages, Human MDMs	Phagosomal damage, Syk activation, Lysosomal permeabilization	EsxA	(11)
*M. marinum*	E11 strain	NLRP3	Human primary Type 1 macrophages	Potassium efflux, ROS production and cathepsin B release	ESX-5	(12)
*M. abscessus*	ATCC 19977	NLRP3	Human MDMs, THP-1 macrophages	dectin-1/Syk-dependent signaling, expression of the cytoplasmic scaffold protein p62/SQSTM1 (p62) and Potassium efflux leads to activation of Caspase-1 and secretion of IL-1β	ND	(13)
*M. kansasii*	ATCC12478	NLRP3	THP-1 macrophages	Potassium efflux, lysosomal acidification, ROS production and cathepsin B release	possibly ESX-1/EsxA	(14)
*M. tuberculosis*	H37Ra	NLRP3	Primary microglia	NF-Kb in signal 1 and P2X7R in signal 2	ND	(15)
*M. tuberculosis*	H37Rv	NLRP3	Ana-1 mouse macrophage cell line	Potassium efflux	LpqH	(16)
*M. marinum*	M-strain	NLRP3	BMDMs	induces Caspase-1 activation	ESX-1	(17)
*M. marinum*	M-strain	NLRP3	Female C57BL/6 (B6) mice and ASC-KO mice	Promotes secretion of IL-1β	ESX-1	(17)
*M. marinum*	M-strain	NLRP3	BMDMs	Promotes secretion of IL-1β and IL-18	ESX-1	(18)
*M. tuberculosis*	H37Rv ATCC 27294	NLRP3	THP-1 macrophages	induces Caspase-1 activation and IL-1β secretion	EsxA	(19)
*M. tuberculosis*	H37Rv	NLRP3	BMDMs/C57Bl/6 mice	Secretion of activated Cathepsin B into the cytosol	EsxA	(20)
*M. abscessus*	ATCC 19977	NLRP3	BMDMs, J774A.1	Induction of mtROS results in increased IL-1β secretion	enhanced cytosolic escape of bacteria	(21)
*M. tuberculosis*	H37Rv ATCC 25618	NLRP3	BMDCs	induces Caspase-1 activation and IL-1β secretion	partially ESX-1–dependent mechanism	(22)
*M. smegmatis*	mc2 155	AIM2	BMDCs	induction of IFN-β	partially ESX-1–dependent mechanism	(23)
*M. fortuitum*	ATCC 6841	AIM2	BMDCs	induction of IFN-β	ND	(23)
*M. kansasii*	ATCC 12478	AIM2	BMDCs	induction of IFN-β	ND	(23)
*M. tuberculosis*	H37Rv ATCC358121	AIM2	Peritoneal macrophages	induces Caspase-1 activation and IL-1β,IL-18 secretion	Mtb genomic DNA	(24)
*M. bovis*	Beijing strain	AIM2	BMDMs, J774A.1	Up-Regulates the mRNA Expression of AIM2 and ASC, requires potassium efflux and mycobacterial internalization but not Reactive Oxygen Species	ND	(25)
*M. ulcerans*	01G897 and 1615	NLRP3/1	BMDMs, hMDM/C57Bl/6 mice	Toxin binding to TLR-2, membrane permeabilization and ROS production	mycolactone	(26)

‘ND’ denotes Not determined. ‘FA’ indicates first author.

## Overview of Mechanisms of Inflammasome Activation and Its Consequences

**Figure 1:**
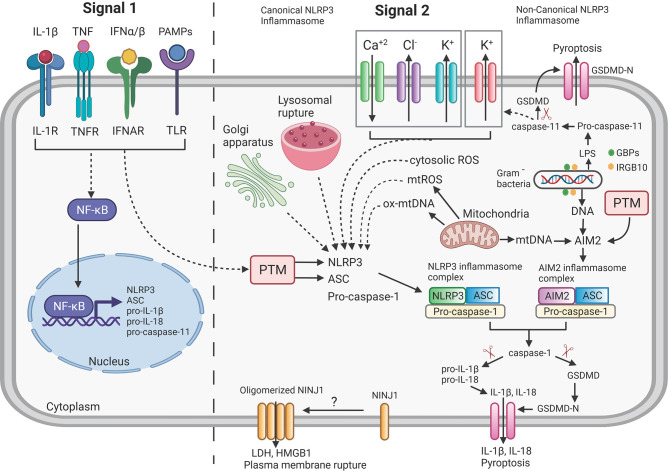
Overview of mechanism of Inflammasome signaling pathway. NLRP3 and AIM2 inflammasome activation requires two distinct signals: Signal 1 (priming signal, left) is induced by the detection of pathogen-associated molecular patterns (PAMPs) or endogenous cytokines by the Toll-like receptor (TLR) or cytokine receptors (IL-1R, TNFR, IFNAR) and thus leading to increased transcription of NLRP3, ASC, pro-IL-1β, pro-IL-18 and pro-caspase-11 through activation of NF-κB. Signal 2 (activation signal, right) for the NLRP3 inflammasome is triggered by various stimuli such as potassium (K^+^) efflux, chloride (Cl^-^) efflux, calcium (Ca^+2^) influx, oxidized mitochondrial DNA (ox-mtDNA), lysosomal rupture and intracellular reactive oxygen species (ROS) production. All these triggers lead to oligomerization and assembly of NLRP3 inflammasome complex. AIM2 directly recognizes either DNA released from Gram negative bacteria or mtDNA released from mitochondria and lead to assembly of AIM2 inflammasome complex. Activated inflammasome complexes (NLRP3 or AIM2) recruit and cleave pro-caspase-1 to active caspase-1 that further results in the proteolytic cleavage of pro-IL-1β and pro-IL-18 to the mature forms IL-1β and IL-18. Caspase-1 also cleaves gasdermin D (GSDMD) to its pore-forming N-terminal fragment GSDMD-N which results in pyroptosis. Ninjurin-1 (NINJ1) by unknown mechanism as indicated by “?” oligomerizes and forms a pore to facilitate release of LDH, HMGB1 and the accumulation of these pores ultimately led to plasma membrane rupture which is not achieved by the GSDMD-N pores. Post-translational modifications (PTM) of cytosolic sensors (NLRP3/AIM2) and adaptor protein (ASC) regulate the activity of inflammasome. Gram negative bacteria are lysed to releases LPS and DNA *via* a mechanism requiring various interferon-inducible Guanylate-binding proteins (GBPs) and IRGB10. The LPS binds to pro-caspase-11 to initiate autocleavage into active caspase-11 which further cleaves GSDMD to GSDMD-N and results in pyroptosis (indicated as Non-Canonical NLRP3 inflammasome) and increased efflux of K^+^, thus further activating the canonical NLRP3 inflammasome pathway (dashed lines = indirect interaction; solid lines = direct interaction; arrowhead = activation). Created with Biorender.com.

In this section we will provide a concise overview of the components and signaling pathways involving mainly two (NLRP3, AIM2) kinds of inflammasomes since they are most relevant to our subsequent discussion on interaction of mycobacteria with inflammasomes (see also **Figure 1**). We would encourage the interested reader to follow-up on this brief overview with any of the excellent specialized reviews on the topic for an in-depth discussion (27–36).

As opposed to the membrane-bound pathogen recognition receptors (PRRs) such as Toll-like receptors (TLRs) that survey extracellular pathogen components, the nucleotide binding and oligomerization domain-like receptors (NLRs, e.g. NLRP3) and Absent in Melanoma 2 (AIM2)-like receptors (ALRs, e.g. AIM2) survey the host cell cytosol for the presence of pathogens (27, 37–41). Upon binding of NLRs/ALRs to pathogen or danger associated molecular patterns (P/DAMP), they initiate the formation the inflammasome (Signal 2: **Figure 1**). IL-1β is a potent immunomodulator and its overproduction may cause rheumatoid arthritis and other pathologies (42, 43). Consequently, IL-1β production is highly regulated and in addition to the inflammasome activation pathway another signaling pathway (Signal 1: **Figure 1**) needs to be engaged to achieve production of mature IL-1β. This pathway involves other PRRs, for example, TLRs or cytokine receptors such as receptors for IL-1β and Tumor Necrosis Factor (TNF) which, after ligand binding, activate NFκB, resulting in the transcriptional activation of, for example, the *IL1B* gene to increase protein levels of pro-IL-1β (44). There is also a crosstalk of Signal 1 with Signal 2 *via* the activation of proteins that perform posttranslational modifications (PTM) of inflammasome components (44–46) (**Figure 1**).

Once Signal 1 and Signal 2 are activated, in case of AIM2 or NLRP3, they associate with the adapter molecule Apoptosis-associated speck-like protein containing a CARD (ASC) which recruits pro-caspase-1 into a complex that continues to oligomerize to form in some cases structures called “specks”, measuring 1-2μm in diameter (47–49). The formed inflammasome complex supports the self-cleavage of pro-caspase-1 into active caspase-1 which cleaves pro-IL-1β and pro-IL-18 and gasdermin D (GSDMD) to release the N-terminal fragment that is capable of oligomerization and membrane pore formation (50–52). The GSDMD pores in the cell membrane will lead to pyroptosis and cytokine release but they do not allow for plasma membrane rupture and secretion of higher molecular weight proteins and protein complexes (e.g., HMGB1) (53) (**Figure 1**). More recently, a complementary role in pore generation after inflammasome activation has been determined for gasdermin E (54).

## Signal 2: Activation of the NLRP3 and AIM2 Inflammasomes

The activation of the NLRP3 inflammasome is complex and one of the reasons is that no ligand that physically binds to NLPR3 has yet been identified. Instead, the prevailing model is that NLRP3 is a stress sensor of the cell which reacts to the increase of various cellular stress signals (32) (**Figure 1**). A major trigger shared across many activating stimuli is the efflux of potassium ions (K^+^) (55). Additional triggers are the efflux of chloride ions (Cl^-^) (56–58), mobilization of intracellular calcium ions (Ca^2+^) (59–61), increase in intracellular reactive oxygen species (ROS) (62) or the release of oxidized mitochondrial DNA (63) [for review (41, 45, 46, 64)] (**Figure 1**). The non-canonical NLRP3 inflammasome pathway (for review (65)) targets caspase-11 in mice and caspases-4/5 in humans (66) and is dependent on the TRIF-mediated induction of interferon (IFN) production and subsequent IFN-receptor mediated signaling (67, 68). Ultimately, the activation of non-canonical pathway is triggered by the presence of intracellular lipopolysaccharide (LPS) of gram-negative bacteria (69, 70) which directly binds to and activates caspases-4/5/11 (71) (**Figure 1**). IFN-stimulated genes, such as the family of guanylate-binding proteins (GBPs), are critical for non-canonical NLRP3 inflammasome activation (72, 73) *via* mechanisms that involve: attacking the outer membrane of cytosolic bacteria such as *Shigella* or *Fransicella* (74–76), releasing intracellular phagosomal bacteria (e.g. *Salmonella*) into the cytosol (73) or assembly to provide a platform for caspase-4 recruitment and activation on the surface of the bacteria (77, 78). Another IFN-stimulated gene product, IRGB10, is also involved in release LPS from intracellular bacteria (75). After activation, caspase-11, like caspase-1, cleaves GSDMD which will lead to pore formation and pyroptosis but not cytokine maturation (51, 52). The activation of the non-canonical pathway will ultimately also lead to efflux of K^+^ which will activate the canonical NLRP3 inflammasome pathway (79) (**Figure 1**).

The signal transduction of the activation of the AIM2 inflammasome is fairly simple because it is mediated by the binding of the HIN-200 domain of AIM2 to DNA (80–82) (**Figure 1**). The pathogen DNA can be accumulating in the cytosol due to infection of the cell with viruses or they can be generated by degrading bacteria in the cytosol *via* action of GBPs and IRGB10 (73–76). Cell stress leading to the release of non-oxidized mitochondrial DNA can also activate the AIM2 inflammasome (83) (**Figure 1**).

Two other important aspects to inflammasome regulation, namely post translational modifications (PTMs) and intracellular location of inflammasome components, will not be discussed in detail here because very little is known about the effect of mycobacterial infection on these two parameters. PTMs in inflammasome activation involves phosphorylation, ubiquitination, sumoylation, S-nitrosylation and ADP-ribosylation of inflammasome components which may lead to either activation or inhibition of the inflammasome formation (44–46, 64, 84). Consequently, the proteins involved in mediating the PTMs are themselves important components of the inflammasome regulatory network. In addition, the subcellular localization of inflammasome components and their association with specific organelles impact activation of the inflammasome (35, 46) (**Figure 1**).

## Inflammasome-Independent Production of IL-1β

It is important to mention that in certain settings mature IL-1β and IL-18 can be generated mostly without the activation of the inflammasome [for review (85, 86)]. The inflammasome-independent IL-1β and IL-18 production is most relevant in an *in vivo* setting where neutrophils dominate (85, 86). The proteases produced in neutrophils involved in cleavage of pro-IL-1β are: proteinase 3 (87, 88), zinc-dependent metalloproteinase meprin A and its monomer meprin α (89), matrix metalloproteinases-2, -3 and -9 (90) and granzyme A (91).

Another pathway for inflammasome independent generation of mature IL-1β and IL-18 is *via* activation of the caspase-8 and its subsequent cleavage of pro-IL-1β and pro-IL-18 (31, 92). One possible pathway for caspase-8 activation is through ligand binding to TLR3 or TLR4 which leads to recruitment of TRIF (Toll/IL-1R domain-containing adapter-inducing IFN-β) and subsequent recruitment of receptor interacting protein 1/receptor interacting protein serine/threonine kinase 1 (RIP1/RIPK1), FAS-associated death domain (FADD) and caspase-8 (93–95). The TNF receptor family member Fas can also activate the FADD/caspase-8 pathway to induce mature IL-1β and IL-18 (96, 97). Other studies also implicated the RIPK3 in the caspase-8 activation pathway (98, 99).

Another inflammasome-independent pathway involving caspase-8 is important for IL-1β and IL-18 production in response to fungal pathogens (100). Dectin-1 signals *via* the tyrosine kinase SYK to induce the formation of a CARD9, Bcl-10, MALT1 and caspase-8 complex which recruits the ASC protein to finally mediate cleavage of pro-IL-1β and -IL-18 (100, 101). Active caspase-8 cleaves pro-IL-1β at the same site that caspase-1 does (93).

## The Impact of IL-1β on Host Response During Mtb Infection

### Protective Role of IL-1β During Mtb Infections

The role of IL-1β throughout infection of the host with Mtb is complex with some evidence in support of a host protective role and other data supporting a role in increasing host susceptibility (3). First, we will discuss the data demonstrating that IL-1β is of importance for host resistance against infections with Mtb and the possible mechanisms (3, 102–106). Mouse studies demonstrate the hyper susceptibility of mice deficient in the expression of either IL-1α/-β or the IL-1β receptor (107–112). The mechanism of protection conferring host resistance by IL-1β has been proposed to involve cell intrinsic mechanisms *via* the increase in host cell apoptosis (113) or autophagy signaling (114). The inflammasome activation has been linked to increasing maturation of Mtb-containing phagosomes and thus limiting bacterial growth (115, 116). Nevertheless, another study demonstrates that cell intrinsic mechanisms are not how IL-1β confers host resistance but instead it is mediated *via trans*-protection of infected cells (117). Although the precise mechanism is unclear*, in vivo*, a major function of IL-1β seems to be to suppress necrosis of lung cells (110, 118). This seems counterintuitive since inflammasome-mediated IL-1β production is associated with cell death (pyroptosis) but it was shown that, at least in the mouse model, IL-1β production is independent of the inflammasome during Mtb infections (111).

It is well-established that, at least partially, the protective effect of IL-1β *in vivo* is linked to its capacity to suppress IFN-β expression since increased IFN-β production increases host susceptibility (for review (3, 102, 104, 106, 119, 120). Importantly, IL-1β can suppress IFN-β expression and vice versa (121). This interdependence can be exploited for HDT approaches to boost IL-1β production, reduce IFN-β expression and reduce the associated morbidities and mortalities (118). Interestingly, IFN-β-mediated signaling is associated with increased necrotic cell death during *ex vivo* Mtb infections which could provide a mechanism for the *in vivo* observed immunopathologies associated with high IFN-β expression (122). Mtb clinical isolates associated with severe TB evade NLRP3 inflammasome activation suggesting a host protective role of IL-1β (123). Macrophages isolated from patients with inflammatory disease carrying gain-of-function genetic variants in inflammasome genes (NLRP3 and/or CARD8) subsequently infected with Mtb displayed increased growth restriction of Mtb in human macrophages (116). In the zebrafish/Mmar infection model, treatment with the drug clemastine modulates the host innate immunity *via* potentiation of P2RX7 that enhances calcium transients within infected macrophages *in vivo*. P2RX7 potentiation augments inflammasome activation, resulting in constraint of mycobacterial growth in zebrafish larvae (124).

### Detrimental Role of IL-1β During Mtb Infections

In contrast, other data from mouse and human studies point towards a role of IL-1β in increasing host susceptibility (3). Some of the strongest evidence for a positive correlation between increased IL-1β and severity of disease in humans comes from several studies analyzing genetic variability and clinical outcomes. The analysis of single nucleotide polymorphisms (SNP) in the human *IL1B* gene identified 3 SNPs in the genes promoter region that results in increased IL-1β expression and was associated with more severe tuberculosis possibly due to the increased infiltration of neutrophils (125). A polymorphism in the IL-1 receptor agonist (*IL1RA*) gene resulted in population with decreased *IL1RA* and increased *IL1B* gene expression that was more commonly found in patients with tuberculoid pleurisy (126). Furthermore, several studies using the mouse model suggest a detrimental role of IL-1β to host defense. For example, it was demonstrated that the primary protective mechanism of nitric oxide (NO) during Mtb infection is not antibacterial activity but instead the suppression of inflammasome activation (127, 128). MCC950 inhibits the NLRP3 inflammasome activation in Mtb-infected BMDMs and results in decreased survival of Mtb in addition to reduced processing of IL-1β (129).

In conclusion, it is very likely that, similar to the situation with TNF and IFN-β (130, 131), also for the production of the IL-1β the Goldilocks principle applies with just the right amount of cytokine being produced in the right context at the right time during infection in order to produce a host protective outcome.

## Inflammasome Recognition of Mycobacteria

### Recognition of Mtb by the NLRP3 Inflammasome

Different mycobacterial species express different proteins and lipids which may affect their recognition by NLR/ALR proteins (132) (**Table 1**). Secretion of proinflammatory mature IL-1β or IL-18 during Mtb infection requires activation of NLRP3 inflammasome (9–11, 17, 19, 22, 133). The activation of the NLRP3 inflammasome after Mtb infection is conserved across various cell types: mouse bone marrow derived macrophages (BMDMs) (4, 5, 8–10, 20), peritoneal exudate macrophages (4, 8), THP-1 human macrophages (5, 7, 8, 10, 11, 19), mouse retinal pigment epithelium (RPE) cells (6), primary microglia (15), Ana-1 mouse macrophage (16), mouse bone marrow derived dendritic cells (BMDCs) (10, 22), J774A.1 mouse macrophages (5), PBMCs (7) and human monocyte derived macrophages (hMDMs) (11) (see also **Table 1**). The adaptor ASC, NLRP3 and caspase-1/11 are required for the secretion of IL-1β in Mtb-infected BMDCs (22). Mtb infection induces increased K^+^ and Cl^-^ efflux but does not affect Ca^2+^ flux (134). The phagosomal and mitochondrial ROS are not involved in the activation of the NLRP3 inflammasome upon Mtb infection but instead it is cytosolic ROS, generated by the xanthine oxidase (XO) (134). Interestingly, plasma membrane damage mediated by ESX-1 system triggers increase in K^+^ efflux, consequently activating the NLRP3 inflammasome and pyroptosis, which permits the spreading of Mtb to neighboring cells (135).

### Recognition of NTM by the NLRP3 Inflammasome

The activation of the NLRP3 inflammasome has also been demonstrated for non-tuberculous mycobacteria (NTM) species including *Mycobacterium marinum* (Mmar) (12, 17), *Mycobacterium abscessus* (Mab) (13, 21) and *Mycobacterium kansasii* (Mkan) (14) and *Mycobacterium ulcerans* (26) (**Table 1**). More specifically, Mab leads to caspase-1 activation and release of IL-1β in human macrophages. Dectin-1/Syk-dependent signaling, increased expression of the cytoplasmic scaffold protein p62/SQSTM1 and potassium efflux are implicated in Mab mediated NLRP3 inflammasome activation (13). Consistently, it has been demonstrated that Mab results in increased production of IL-1β in murine macrophages (21). Mab induces mitochondrial ROS and thereby leads to enhanced NLRP3 inflammasome activation (21). A recent study has demonstrated that infection of microglia with the attenuated Mtb H37Ra strain triggers NLRP3 mediated secretion of IL-1β and IL-18 (15). Moreover, they also found that by inhibiting NF-κB (signal 1) and P2X7R (signal 2) they can alter the secretion of IL-1β and IL-18 and thereby regulate the NLRP3 inflammasome pathway in microglia during Mtb infection (15) (**Table 1**). Interestingly, irrespective of the overexpression of NLRP3 and inflammatory caspases-4/5 detected in the lepromatous pole, low expression of caspase-1, IL-1β, and IL-18 were observed in leprosy and therefore these results indicate that NLRP3 inflammasome does not actively contribute to the innate immune response in leprosy, suggesting immune evasion of *M. leprae* (136).

### Recognition of Mycobacteria by the AIM2 Inflammasome

In addition to NLRP3 inflammasome, different reports have implicated the critical role for the AIM2 inflammasome following mycobacterial infection. Activation of AIM2 inflammasome has been reported in several cell types during infection with various mycobacterial species, including *Mycobacterium smegmatis* (Msme) (23)*, Mycobacterium fortuitum* (Mfor) and Mkan in BMDCs (23) (**Table 1**). In BMDCs about 40–50% of the production of IL-1β following Msme, Mfor, and Mkan infection was dependent on the AIM2 inflammasome and moreover there was an inverse correlation between virulence of the mycobacterial species and the amount of IL-1β release, with the least virulent species inducing the highest levels of IL-1β (23). *Mycobacterium bovis* (Mbov) infection activated the AIM2 inflammasome in BMDMs and J774A.1 mouse macrophage (25) (**Table 1**). Mbov infection augments the mRNA expression of AIM2 and ASC in both BMDMs and J774A.1 mouse macrophage (25). Potassium efflux and mycobacterial escape into the cytosol are the two essential triggers in the activation of AIM2 inflammasome during Mbov infection. Additionally, infection of J774A.1 mouse macrophage with Mbov results in activation of caspase-1 as early as 6h post-infection (25). The transfection of Mtb genomic DNA into LPS-primed peritoneal macrophages results in AIM2-dependent caspase-1 activation and subsequent secretion of mature IL-1β and IL-18 (24). This is not surprising since AIM2 recognizes any type of dsDNA but interesting because Mtb does not activate the AIM2 inflammasome upon infection of BMDCs or BMDMs (23).

## Activation of the NLRP3 Inflammasome by Mycobacterial Proteins and Lipids

**Figure 2:**
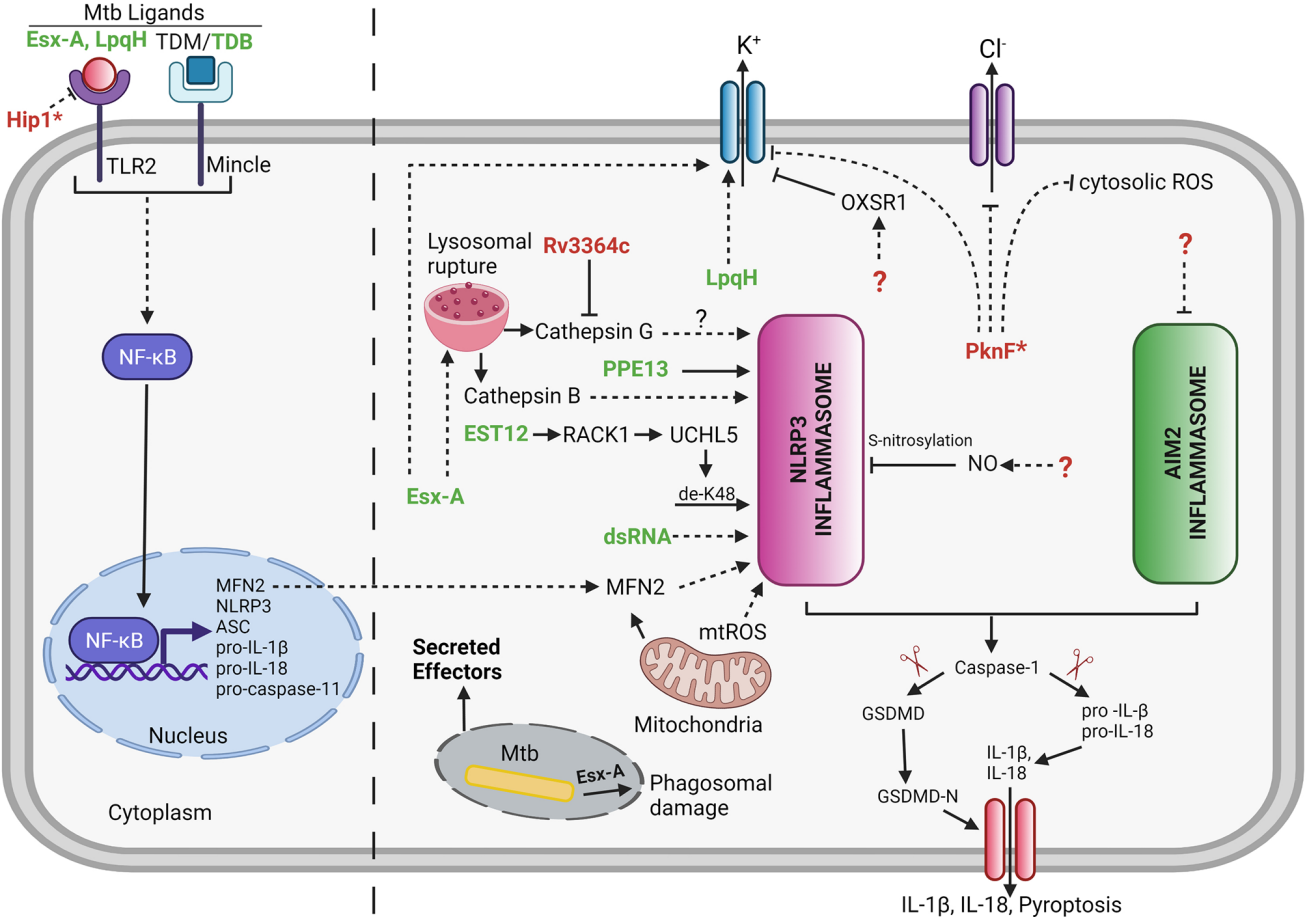
Mycobacterial effectors involved in regulation of host cell inflammasome. Several Mtb effectors either secreted or non-secreted (as indicated by *) are known to be implicated in manipulation of the host cell inflammasome pathway. Bold green color denotes the Mtb effectors involved either directly or indirectly in activation of inflammasome. Bold red color denotes the Mtb effectors involved in inhibition of inflammasome. Unknown Mtb effectors are represented by ? (dashed lines = indirect interaction; solid lines = direct interaction; arrowhead = activation; blunt end = inhibition). LpqH,19 kDa Lipoprotein antigen precursor; PPE13, PPE family protein 13; EST12, Estimated 12kDa (Rv1579c); EsxA, 6 kDa Early secretory antigenic target; dsRNA, double stranded Ribonucleic Acid; TDB, Trehalose-6,6-dibehenate; TDM, Trehalose dimycolate; PknF, Protein kinase F; Hip1, Hydrolase important for pathogenesis 1; NO, Nitric Oxide; RACK1, Receptor for Activated C Kinase 1; UCHL5, Ubiquitin C-Terminal Hydrolase L5; MFN2, Mitofusin 2; OXSR1, Oxidative Stress Responsive Kinase 1. Created with Biorender.com.

Mtb contains several secretion systems to export proteins into the cell well and beyond (137, 138). In order for these proteins to potentially reach the host cell cytosol, the ESX-1-secreted effector EsxA is involved in permeabilizing the phagosomal membrane (**Figure 2**) (139, 140). A small number of mycobacterial secreted protein effectors have been identified that are involved in activation of NLRP3 inflammasome (5–8, 10, 11, 16, 19, 20) (**Figure 2** and **Table 1**).

### PPE13

The Proline-Proline-Glutamate (PPE) family protein, PPE13, participates in the assembly of NLRP3 inflammasome complex *via* its C-terminal repetitive major polymorphic tandem repeat (MPTR) domain by directly interacting with the NACHT and Leucine-rich repeat (LRR) domains of NLRP3 (5) (**Figure 2** and **Table 1**). A recombinant Msme strain expressing the Mbov PPE13 induces increased cell death and increased secretion of IL-1β in J774A.1, BMDMs, and THP-1 macrophages but to different levels depending on the cell type (5). The release of IL-1β was dependent on activation of NLRP3 inflammasome as confirmed by caspase-1 and NLRP3 inflammasome inhibitor studies (5). The role of PPE13 in inflammasome signaling needs further validation by creating a gene specific knockout in Mtb.

### EST12

Recent studies show that *Rv1579c*, located within the Mtb H37Rv region of difference 3 (RD3), encodes for a protein (EST12) which acts as a pyroptosis-inducing protein (8) (**Figure 2** and **Table 1**). Indeed, EST12 interacts with the host protein receptor for activated C kinase 1 (RACK1) and forms a EST12-RACK1 complex in macrophages. The EST12-RACK1 dimer recruits the deubiquitinase UCHL5 to stimulate the K48-linked deubiquitination of NLRP3 and consequently triggers the NLRP3 inflammasome mediated pyroptosis and IL-1β secretion (8) (**Figure 2**). Mice infected with an Mtb strain lacking EST12 showed significant increase in the bacterial growth in the lungs and lower levels of serum IL-1β compared to wild-type Mtb-infected mice. Consistently, mice infected with BCG or Msme strains overexpressing EST12 showed lower bacterial burden in lungs or spleen and increased levels of IL-1β compared to mice infected with control bacteria. Consequently, Mtb EST12 increases mycobacterial clearance in mice and is responsible to activate the host’s immunity (8). It will be interesting to see in future studies which Mtb secretion system is responsive for the secretion of EST12.

### LpqH

The Mtb lipoprotein, LpqH, activates the NLRP3 inflammasome *via* a mechanism that involves activation of the TLR-2 receptor (16) (**Figure 2** and **Table 1**). The treatment of LPS-primed Ana-1 mouse macrophages with purified LpqH protein results in increased expression of NLRP3, ASC and caspase-1 proteins in a dose-dependent manner (16). Moreover, potassium efflux acts as an important trigger for LpqH-mediated activation of the NLRP3 inflammasome (16). However, the underlying mechanism of how LpqH influences potassium efflux has not been explored in this study. The work was performed in mouse macrophages and thus further validation in human macrophages would be valuable. It will also be crucial to test a *lpqH* deletion mutant of Mtb for changes in NLRP3 inflammasome activation to confirm that the observed activity of purified proteins is conserved within the context of a whole bacterium.

### ESX-1 and ESX-5

The Mtb RD1 locus which encodes for the ESX-1 secretion system is required for activation of NLRP3 inflammasome and subsequent release of IL-1β in human PBMCs, THP-1 cells and mouse BMDMs and retinal pigment epithelium (RPE) cells (4, 6, 7, 9, 11, 14, 19, 20) (**Figure 2** and **Table 1**). The ESX-5a is a duplicated region of 4 genes out of the ESX-5 secretion system which is important for the secretion of subset of ESX-5-secreted proteins and its deletion results in reduced inflammasome activation (10) (**Table 1**).

### EsxA

EsxA is one of the main substrates secreted by the ESX-1 system and consequently many studies have been performed by adding purified EsxA to cells during *ex vivo* experimentations. EsxA interacts with TLR-2 and TLR-4 receptors to induce host cell signaling (141–144). One report shows that treatment of mouse RPE cells with different doses of EsxA results in caspase-1 activation in a dose dependent manner and that this activation is dependent on TLR/MyD88 signaling and the NLRP3 inflammasome (6). Other studies reveal that stimulation of either PBMCs (7) or THP-1 macrophages (7, 19) with EsxA results in increased release of proinflammatory cytokine IL-1β. Furthermore, stimulation of PBMCs and THP-1 derived macrophages with either Mtb protein EsxA or heat inactivated Mtb lysates induces increase in expression of MFN2 and results in increased release of IL-1β. Therefore, these findings suggest that MFN2 is required for the assembly and activation of NLRP3 inflammasome during Mtb infection (7). Transcriptional profiling in human PBMCs from active tuberculosis patients and healthy controls determined that a mitochondrial outer membrane protein, MFN2 expression was significantly upregulated in TB patients compared to healthy controls (7). Intriguingly, another study reported the importance of EsxA and RD1 locus in secretion of IL-1β, since BMDMs when infected with Mtb strains lacking *esxA* or RD1 showed a significant reduction in the secretion of IL-1β compared to the BMDMs infected with Mtb (20). EsxA was partially responsible for the release of mature cathepsin B by the lysosomes during Mtb infection and further leads to NLRP3 inflammasome activation in BMDMs (20). In addition to lysosomal permeabilization, EsxA also triggers phagosome damage and Syk activation in human macrophages that result in NLRP3 mediated necrotic cell death (11). Additionally, EsxA facilitates the translocation of other immunostimulatory Mtb components such as Ag85 into the macrophage cytosol, resulting in increased activation of caspase-1 and subsequent secretion of IL-1β (19).

Critical role for the ESX-1/EsxA in NLRP3 activation has also been reported during the infection with non-tuberculous mycobacteria species including Mmar (12, 17, 18), and Mkan (14) (**Table 1**). In addition to ESX-1, ESX-5 secreted substrates also play a role in activation of NLRP3 inflammasome in response to infection with Mmar (12) (**Table 1**). Additionally, transfection of mouse RPE cells with mycobacterial dsRNA induces NLRP3 inflammasome-dependent caspase-1 activation *via* an uncharacterized mechanism (6).

### TDB/TDM

The role of mycobacterial cell wall lipids in NLRP3 inflammasome activation has also been studied in addition to mycobacterial secreted effector proteins (145). Trehalose-6,6’-dibehenate (TDB), a synthetic analogue of Trehalose-6,6’-dimycolate (TDM) also known as mycobacterial cord factor has been developed as an effective adjuvant for tuberculosis subunit vaccine and both act as a potent proinflammatory pathogen-associated molecular pattern (PAMP) which is recognized by macrophage inducible C-type lectin (Mincle) receptor on innate immune cells and triggers host innate immune response. TDB induces the NLRP3/ASC/Caspase-1 mediated increase in production of IL-1β in BMDCs. Activation of NLRP3 inflammasome by TDB involves numerous triggers such as lysosomal permeabilization, increased ROS generation and increased potassium efflux (145).

It is important to remember that the NLRP3 inflammasome is acting most likely as the cells stress or danger sensor and thus any bacterial induction of the NRLP3 inflammasome might not be mediated by a direct interaction with the inflammasome complex but through interaction with other cellular components/pathways that then trigger the stress/danger signal.

## AIM2 Inflammasome Inhibition by Mtb

Mycobacterial extracellular DNA enters the host cell cytosol in an ESX-1 secretion system dependent manner (146). Intriguingly, AIM2 recognizes and binds to cytosolic DNA of intracellular pathogens such as *Francisella* and *Listeria* (82, 147) and even Mtb (24). However, AIM2 is not activated during the course of *ex vivo* Mtb H37Rv infection of BMDM and BMDCs (23). Nonvirulent mycobacterial species such as Msme induce the activation of AIM2 inflammasome in contrast to virulent Mtb that inhibits the AIM2 inflammasome activation induced by either Msme or AIM2 agonists (23). Moreover, Mtb-mediated AIM2 inflammasome inhibition is dependent on a functional ESX-1 secretion system since infection with the Mtb strain deficient in *esxA* fails to inhibit IL-1β secretion induced by Msme (23). Intriguingly, Mtb inhibits the secretion of IFN-β in infected cells, which may provide one of the mechanisms to suppresses the activation of AIM2 inflammasome (23). Consequently, co-secretion of a putative AIM2 inhibitor and/or IFN-β inhibitor through ESX-1 secretion system into the host cell cytosol together with cytosolic Mtb DNA may play an important role in Mtb-mediated evasion of AIM2 inflammasome activation. The inhibition of the AIM2 inflammasome activation might be of importance for virulence of Mtb because *aim2* ^-/-^ mice are highly susceptible to Mtb infections and showing impaired production of pro-inflammatory cytokines IL-1β and IL-18 (24). An area of interest will be the discovery of the Mtb genes involved in the AIM2 inflammasome inhibition in order to assess the importance of AIM2-inflammasoem evasion for the virulence of Mtb.

## NLRP3 Inflammasome Inhibition by Mtb

### Mtb PknF

As discussed before many studies have shown that Mtb causes NLRP3 inflammasome activation and even that the deletion of certain Mtb genes led to an increased activation of the NLRP3 inflammasome (**Figure 2** and **Table 1**). Nevertheless, not until recently was it shown that Mtb infection can inhibit activation of the NLRP3 inflammasome *via* either LPS/Nigericin or LPS/ATP stimuli (134). Mtb inhibits the NLRP3 inflammasome activation *via* a mechanism that is independent of the ESX-1 secretion system which is opposed to the capacity of Mtb to inhibit the AIM2 inflammasome in an ESX-1 dependent mechanism (23, 134). Mtb infection inhibits the LPS/ATP-induced K^+^ efflux and increase in xanthine oxidase (XO) activity leading to decreased cytosolic ROS levels (134). The Mtb serine threonine kinase, PknF, mediates inhibition of NLRP3 inflammasome dependent production of IL-1β and pyroptosis in both mouse and human derived primary macrophages (134). Moreover, K^+^ efflux, Cl^2+^ efflux and ROS generation are implicated in the PknF-mediated NLRP3 inflammasome inhibition (134) (**Figure 2**). Additionally, the Mtb *pknF* mutant induces an increase in the XO activity compared to Mtb-infected cells and thus increasing XO-mediated ROS production (134). Altogether it seems that PknF is inhibiting the exact NLRP3 inflammasome activation pathway that is being only slightly activated upon Mtb infection.

### Mtb Zmp1

The BCG gene *zmp1* encodes a zinc metalloprotease that has been shown to inhibit the NLRP3 inflammasome dependent processing of IL-1β (115). However, these findings could not be independently confirmed since generation of the *zmp1* Mtb deletion mutant strain did not show any effect on pyroptosis, on the caspase-1 activation nor the release of IL-1β (11). The inconsistency between these two publications might be due to a difference in the background of the *zmp1* mutant strain because most of the studies performed by Master et al. were done using the BCG strain that, unlike Mtb, lacks a functional ESX-1 secretion system. Another possibility may be the difference in cell types used to study the strains lacking *zmp1*.

### Mtb Hip1 and Rv3364c

The Mtb serine hydrolase, Hip1, inhibits the NLRP3 inflammasome activation by dampening the TLR2-dependent cell signaling in BMDMs (148) (**Figure 2**). Hip1 not only affects the secretion of inflammasome dependent cytokines but also result in decrease of other proinflammatory cytokines thus suggesting Hip1 modulates the proinflammatory responses in macrophages by preventing the activation of TLR2 dependent cell signaling (148). The mechanism involves Hip1-mediated proteolytical cleavage of GroEL2 from multimer to monomer and the cleaved monomeric GroEL2 subsequently contributes to dampening of proinflammatory responses in macrophages mediated by TLR2 signaling (149). Therefore, it is not possible to attribute the *in vivo* attenuation of the *hip1* Mtb deletion mutant to its increase in inflammasome activation since also other important proinflammatory cytokines such as TNF are upregulated (148).The Mtb protein, Rv3364c, binds to and inhibits the membrane associated host serine protease cathepsin G which leads to suppression of caspase-1 activity and pyroptosis in macrophages (150) (**Figure 2**).

### Nitric Oxide (NO)

Host cell derived NO acts as a negative regulator of NLRP3 inflammasome activation and inhibits processing of IL-1β (127, 151) (**Figure 2**). Stimulation of Mtb-infected macrophages with IFN-γ showed iNOS-dependent thiol nitrosylation of NLRP3 which leads to inhibition of NLRP3 inflammasome dependent maturation of IL-1β and minimize inflammatory tissue damage during chronic Mtb infection (127). Indeed, the NO-mediated nitrosylation inhibits the assembly of NLRP3 inflammasome complex (127, 151).

### OXSR1

During mycobacterial infection, a host serine/threonine protein kinase, the oxidative stress responsive kinase 1 (OXSR1), inhibits K^+^ channels responsible for K^+^ efflux (152). Indeed, the mycobacterial infection leads to the upregulation of host OXSR1 and this host cell manipulation is dependent on the mycobacterial ESX-1 secretion system. The immunomodulatory role of OXSR1 is conserved in both zebrafish and humans (152). Furthermore, inhibition or depletion of OXSR1 results in diminished levels of intracellular potassium and hence limits the growth of mycobacteria. Therefore, targeting of OXSR1 might be a valuable approach for host-directed therapy. Micheliolide (MCL), a sesquiterpene lactone, act as an anti-inflammatory molecule by inhibiting PI3K/Akt/NF-κB and NLRP3 inflammasome signaling during Mtb infection (153). However, previous report showed that RAW264.7 cells do not release mature IL-1β since they do not express ASC as determined by immunoblot analysis with an ASC-specific antibody (154). The study by Zhang et al. did not address this problem since all experiments were performed in RAW264.7 macrophages and hence there remains some questions on the validity of their results.

Overall, there has been notable progress but still the molecular mechanisms by which Mtb evades NLRP3 inflammasome activation and the importance of this manipulation for virulence of Mtb remain poorly understood.

## Conclusion

Since we last reviewed the literature on the subject of Mtb-host cell inflammasome interactions in 2013 (155) a tremendous amount of progress has been made in our understanding of the molecular mechanisms of inflammasome activation and subsequent pathways of pyroptosis induction (**Figure 1**). Also, our knowledge of the role of IL-1β during Mtb infections has been greatly expended. Nevertheless, important questions remain; for example, what is the *in vivo* mechanisms of host resistance that is mediated by IL-1β? We know that protective effects are mediated by bystander cells but what is IL-1β/IL-1β-signaling doing onto those cells that conveys the protective effect? We now know that Mtb is able to inhibit the AIM2- and NLRP3-inflammasome during *ex vivo* infections (**Figure 2**) but is there a role for the inflammasome for increasing host resistance or susceptibility during *in vivo* Mtb infections? The herein described progress made in identifying various Mtb effectors activating or inhibiting the host cell inflammasome (**Figure 2** and **Table 1**) and the subsequent availability of specific Mtb mutants perturbing the inflammasome activation will provide the tools necessary to start answering that question.

## Author Contributions

SR and VB wrote and edited the manuscript. SR created the figures and table. All authors contributed to the article and approved the submitted version.

## Funding

SR and VB are funded by NIH/NIAID grants AI139492 and AI147630.

## Conflict of Interest

The authors declare that the research was conducted in the absence of any commercial or financial relationships that could be construed as a potential conflict of interest.

## Publisher’s Note

All claims expressed in this article are solely those of the authors and do not necessarily represent those of their affiliated organizations, or those of the publisher, the editors and the reviewers. Any product that may be evaluated in this article, or claim that may be made by its manufacturer, is not guaranteed or endorsed by the publisher.

## References

[1] WHO. Global Tuberculosis Report 2020 (2020). Available at: https://www.who.int/teams/global-tuberculosis-programme/tb-reports/global-tuberculosis-report-2020.

[2] Pai M Behr MA Dowdy D Dheda K Divangahi M Boehme CC. Tuberculosis. Nat Rev Dis Primers (2016) 2:16076. doi: 10.1038/nrdp.2016.76 27784885

[3] Mayer-Barber KD Sassetti CM. Type I Interferon and Interleukin-1 Driven Inflammatory Pathways as Targets for HDT in Tuberculosis. In: Karakousis PC Hafner R Gennaro ML, editors. Advances in Host-Directed Therapies Against Tuberculosis. Springer International Publishing (2021). p. 219–32.

[4] Kurenuma T Kawamura I Hara H Uchiyama R Daim S Dewamitta SR. The RD1 Locus in the Mycobacterium Tuberculosis Genome Contributes to Activation of Caspase-1 via Induction of Potassium Ion Efflux in Infected Macrophages ▿. Infect Immun (2009) 77:3992–4001. doi: 10.1128/iai.00015-09 19596775 PMC2737993

[5] Yang Y Xu P He P Shi F Tang Y Guan C. Mycobacterial PPE13 Activates Inflammasome by Interacting With the NATCH and LRR Domains of NLRP3. FASEB J (2020) 34:12820–33. doi: 10.1096/fj.202000200rr 32738179

[6] Basu S Fowler BJ Kerur N Arnvig KB Rao NA. NLRP3 Inflammasome Activation by Mycobacterial ESAT-6 and Dsrna in Intraocular Tuberculosis. Microb Pathogen (2018) 114:219–24. doi: 10.1016/j.micpath.2017.11.044 29180292

[7] Xu F Qi H Li J Sun L Gong J Chen Y. Mycobacterium Tuberculosis Infection Up-Regulates MFN2 Expression to Promote NLRP3 Inflammasome Formation. J Biol Chem (2020) 295:17684–97. doi: 10.1074/jbc.ra120.014077 PMC7762945 33454007

[8] Qu Z Zhou J Zhou Y Xie Y Jiang Y Wu J. Mycobacterial EST12 Activates a RACK1–NLRP3–Gasdermin D Pyroptosis–IL-1β Immune Pathway. Sci Adv (2020) 6:eaba4733. doi: 10.1126/sciadv.aba4733 33097533 PMC7608829

[9] Dorhoi A Nouailles G Jörg S Hagens K Heinemann E Pradl L. Activation of the NLRP3 Inflammasome by Mycobacterium Tuberculosis is Uncoupled From Susceptibility to Active Tuberculosis. Eur J Immunol (2012) 42:374–84. doi: 10.1002/eji.201141548 22101787

[10] Shah S Cannon JR Fenselau C Briken V. A Duplicated ESAT-6 Region of ESX-5 is Involved in Protein Export and Virulence of Mycobacteria. Infect Immun (2015) 83:4349–61. doi: 10.1128/iai.00827-15 PMC4598393 26303392

[11] Wong K Jr WRJ. Critical Role for NLRP3 in Necrotic Death Triggered by Mycobacterium Tuberculosis. Cell Microbiol (2011) 13:1371–84. doi: 10.1111/j.1462-5822.2011.01625.x PMC3257557 21740493

[12] Abdallah AM Bestebroer J Savage NDL de Punder KZ van M Wilson L. Mycobacterial Secretion Systems ESX-1 and ESX-5 Play Distinct Roles in Host Cell Death and Inflammasome Activation. J Immunol (2011) 187:4744–53. doi: 10.4049/jimmunol.1101457 21957139

[13] Lee H Yuk J Kim K Jang J Kang G Park JB. Mycobacterium Abscessus Activates the Nlrp3 Inflammasome via Dectin-1–Syk and P62/Sqstm1. Immunol Cell Biol (2012) 90:601–10. doi: 10.1038/icb.2011.72 PMC3389799 21876553

[14] Chen C-C Tsai S-H Lu C-C Hu S-T Wu T-S Huang T-T. Activation of an NLRP3 Inflammasome Restricts Mycobacterium Kansasii Infection. PloS One (2012) 7:e36292. doi: 10.1371/journal.pone.0036292 22558425 PMC3340363

[15] Xie Z Hui H Yao Q Duan Y Li W Cheng Y. By Regulating the NLRP3 Inflammasome can Reduce the Release of Inflammatory Factors in the Co-Culture Model of Tuberculosis H37Ra Strain and Rat Microglia. Front Cell Infect Mi (2021) 11:637769:637769. doi: 10.3389/fcimb.2021.637769 PMC8078893 33928044

[16] Liu L Zhai K Chen Y Chen X Wang G Wu L. Effect and Mechanism of Mycobacterium Tuberculosis Lipoprotein Lpqh in NLRP3 Inflammasome Activation in Mouse Ana-1 Macrophage. BioMed Res Int (2021) 2021:1–8. doi: 10.1155/2021/8239135 PMC7803426 33490276

[17] Carlsson F Kim J Dumitru C Barck KH Carano RAD Sun M. Host-Detrimental Role of Esx-1-Mediated Inflammasome Activation in Mycobacterial Infection. PloS Pathog (2010) 6:e1000895. doi: 10.1371/journal.ppat.1000895 20463815 PMC2865529

[18] Koo IC Wang C Raghavan S Morisaki JH Cox JS Brown EJ. ESX-1-Dependent Cytolysis in Lysosome Secretion and Inflammasome Activation During Mycobacterial Infection. Cell Microbiol (2008) 10:1866–78. doi: 10.1111/j.1462-5822.2008.01177.x PMC2574867 18503637

[19] Mishra BB Moura-Alves P Sonawane A Hacohen N Griffiths G Moita LF. Mycobacterium Tuberculosis Protein ESAT-6 is a Potent Activator of the NLRP3/ASC Inflammasome. Cell Microbiol (2010) 12:1046–63. doi: 10.1111/j.1462-5822.2010.01450.x 20148899

[20] Amaral EP Riteau N Moayeri M Maier N Mayer-Barber KD Pereira RM. Lysosomal Cathepsin Release is Required for NLRP3-Inflammasome Activation by Mycobacterium Tuberculosis in Infected Macrophages. Front Immunol (2018) 9:1427. doi: 10.3389/fimmu.2018.01427 29977244 PMC6021483

[21] Kim B-R Kim B-J Kook Y-H Kim B-J. Mycobacterium Abscessus Infection Leads to Enhanced Production of Type 1 Interferon and NLRP3 Inflammasome Activation in Murine Macrophages via Mitochondrial Oxidative Stress. PloS Pathog (2020) 16:e1008294. doi: 10.1371/journal.ppat.1008294 32210476 PMC7094820

[22] Abdalla H Srinivasan L Shah S Mayer-Barber KD Sher A Sutterwala FS. Mycobacterium Tuberculosis Infection of Dendritic Cells Leads to Partially Caspase-1/11-Independent IL-1β and IL-18 Secretion But Not to Pyroptosis. PloS One (2012) 7:e40722. doi: 10.1371/journal.pone.0040722 22911706 PMC3404059

[23] Shah S Bohsali A Ahlbrand SE Srinivasan L Rathinam VAK Vogel SN. Cutting Edge: Mycobacterium Tuberculosis But Not Nonvirulent Mycobacteria Inhibits IFN-B and AIM2 Inflammasome–Dependent IL-1β Production via its ESX-1 Secretion System. J Immunol (2013) 191:3514–8. doi: 10.4049/jimmunol.1301331 PMC3799997 23997220

[24] Saiga H Kitada S Shimada Y Kamiyama N Okuyama M Makino M. Critical Role of AIM2 in Mycobacterium Tuberculosis Infection. Int Immunol (2012) 24:637–44. doi: 10.1093/intimm/dxs062 22695634

[25] Yang Y Zhou X Kouadir M Shi F Ding T Liu C. The AIM2 Inflammasome is Involved in Macrophage Activation During Infection With Virulent Mycobacterium Bovis Strain. J Infect Dis (2013) 208:1849–58. doi: 10.1093/infdis/jit347 23901081

[26] Foulon M Robbe-Saule M Manry J Esnault L Boucaud Y Alcaïs A. Mycolactone Toxin Induces an Inflammatory Response by Targeting the IL-1β Pathway: Mechanistic Insight Into Buruli Ulcer Pathophysiology. PloS Pathog (2020) 16:e1009107. doi: 10.1371/journal.ppat.1009107 33338061 PMC7748131

[27] Rathinam VAK Fitzgerald KA. Inflammasome Complexes: Emerging Mechanisms and Effector Functions. Cell (2016) 165:792–800. doi: 10.1016/j.cell.2016.03.046 27153493 PMC5503689

[28] Sharma D Kanneganti T-D. The Cell Biology of Inflammasomes: Mechanisms of Inflammasome Activation and Regulation. J Cell Biol (2016) 213:617–29. doi: 10.1083/jcb.201602089 PMC4915194 27325789

[29] Man SM Karki R Kanneganti T-D. Molecular Mechanisms and Functions of Pyroptosis, Inflammatory Caspases and Inflammasomes in Infectious Diseases. Immunol Rev (2017) 277:61–75. doi: 10.1111/imr.12534 28462526 PMC5416822

[30] Broz P. Recognition of Intracellular Bacteria by Inflammasomes. Microbiol Spectr (2019) 7. doi: 10.1128/microbiolspec.bai-0003-2019 PMC11588290 30848231

[31] Chan AH Schroder K. Inflammasome Signaling and Regulation of Interleukin-1 Family Cytokines. J Exp Med (2019) 217:e20190314. doi: 10.1084/jem.20190314 PMC7037238 31611248

[32] Evavold CL Kagan JC. Inflammasomes: Threat-Assessment Organelles of the Innate Immune System. Immunity (2019) 51:609–24. doi: 10.1016/j.immuni.2019.08.005 PMC6801093 31473100

[33] Broz P Pelegrín P Shao F. The Gasdermins, a Protein Family Executing Cell Death and Inflammation. Nat Rev Immunol (2020) 20:143–57. doi: 10.1038/s41577-019-0228-2 31690840

[34] Linder A Hornung V. Irgm2 and Gate-16 Put a Break on non-Canonical Inflammasome Activation. EMBO Rep (2020) 11:561948–3. doi: 10.15252/embr.202051787 PMC7645257 33135287

[35] Seoane PI Lee B Hoyle C Yu S Lopez-Castejon G Lowe M. The NLRP3–Inflammasome as a Sensor of Organelle Dysfunction. J Cell Biol (2020) 219:e202006194. doi: 10.1083/jcb.202006194 33044555 PMC7543090

[36] Deets KA Vance RE. Inflammasomes and Adaptive Immune Responses. Nat Immunol (2021) 22:412–22. doi: 10.1038/s41590-021-00869-6 33603227

[37] Lamkanfi M Dixit VM. Inflammasomes: Guardians of Cytosolic Sanctity. Immunol Rev (2009) 227:95–105. doi: 10.1111/j.1600-065x.2008.00730.x 19120479

[38] Broz P Monack DM. Molecular Mechanisms of Inflammasome Activation During Microbial Infections. Immunol Rev (2011) 243:174–90. doi: 10.1111/j.1600-065x.2011.01041.x PMC3170129 21884176

[39] Bauernfeind F Hornung V. Of Inflammasomes and Pathogens–Sensing of Microbes by the Inflammasome. EMBO Mol Med (2013) 5:814–26. doi: 10.1002/emmm.201201771 PMC3779445 23666718

[40] Moltke J Ayres JS Kofoed EM Chavarría-Smith J Vance RE. Recognition of Bacteria by Inflammasomes. Annu Rev Immunol (2013) 31:73–106. doi: 10.1146/annurev-immunol-032712-095944 23215645

[41] Hayward JA Mathur A Ngo C Man SM. Cytosolic Recognition of Microbes and Pathogens: Inflammasomes in Action. Microbiol Mol Biol R (2018) 82:e00015–18. doi: 10.1128/mmbr.00015-18 PMC6298609 30209070

[42] Garlanda C Dinarello CA Mantovani A. The Interleukin-1 Family: Back to the Future. Immunity (2013) 39:1003–18. doi: 10.1016/j.immuni.2013.11.010 PMC3933951 24332029

[43] Jesus AA Goldbach-Mansky R. IL-1 Blockade in Autoinflammatory Syndromes1. Annu Rev Med (2014) 65:223–44. doi: 10.1146/annurev-med-061512-150641 PMC4178953 24422572

[44] McKee CM Coll RC. NLRP3 Inflammasome Priming: A Riddle Wrapped in a Mystery Inside an Enigma. J Leukocyte Biol (2020) 108:937–52. doi: 10.1002/jlb.3mr0720-513r 32745339

[45] Yang Y Wang H Kouadir M Song H Shi F. Recent Advances in the Mechanisms of NLRP3 Inflammasome Activation and its Inhibitors. Cell Death Dis (2019) 10:128. doi: 10.1038/s41419-019-1413-8 30755589 PMC6372664

[46] Weber ANR Bittner ZA Shankar S Liu X Chang T-H Jin T. Recent Insights Into the Regulatory Networks of NLRP3 Inflammasome Activation. J Cell Sci (2020) 133:jcs248344. doi: 10.1242/jcs.248344 33273068

[47] Fernandes-Alnemri T Wu J Yu J-W Datta P Miller B Jankowski W. The Pyroptosome: A Supramolecular Assembly of ASC Dimers Mediating Inflammatory Cell Death via Caspase-1 Activation. Cell Death Differ (2007) 14:1590–604. doi: 10.1038/sj.cdd.4402194 PMC3345951 17599095

[48] Cai X Chen J Xu H Liu S Jiang Q-X Halfmann R. Prion-Like Polymerization Underlies Signal Transduction in Antiviral Immune Defense and Inflammasome Activation. Cell (2014) 156:1207–22. doi: 10.1016/j.cell.2014.01.063 PMC4034535 24630723

[49] Lu A Magupalli VG Ruan J Yin Q Atianand MK Vos MR. Unified Polymerization Mechanism for the Assembly of ASC-Dependent Inflammasomes. Cell (2014) 156:1193–206. doi: 10.1016/j.cell.2014.02.008 PMC4000066 24630722

[50] He W Wan H Hu L Chen P Wang X Huang Z. Gasdermin D is an Executor of Pyroptosis and Required for Interleukin-1β Secretion. Cell Res (2015) 25:1285–98. doi: 10.1038/cr.2015.139 PMC4670995 26611636

[51] Kayagaki N Stowe IB Lee BL O’Rourke K Anderson K Warming S. Caspase-11 Cleaves Gasdermin D for non-Canonical Inflammasome Signalling. Nature (2015) 526:666–71. doi: 10.1038/nature15541 26375259

[52] Shi J Zhao Y Wang K Shi X Wang Y Huang H. Cleavage of GSDMD by Inflammatory Caspases Determines Pyroptotic Cell Death. Nature (2015) 526:660–5. doi: 10.1038/nature15514 26375003

[53] Kayagaki N Kornfeld OS Lee BL Stowe IB O’Rourke K Li Q. NINJ1 Mediates Plasma Membrane Rupture During Lytic Cell Death. Nature (2021), 591(7848):131–6. doi: 10.1038/s41586-021-03218-7 33472215

[54] Zhou B Abbott DW. Gasdermin E Permits Interleukin-1 Beta Release in Distinct Sublytic and Pyroptotic Phases. Cell Rep (2021) 35:108998. doi: 10.1016/j.celrep.2021.108998 33852854 PMC8106763

[55] Muñoz-Planillo R Kuffa P Martínez-Colón G Smith BL Rajendiran TM Núñez G. K+ Efflux is the Common Trigger of NLRP3 Inflammasome Activation by Bacterial Toxins and Particulate Matter. Immunity (2013) 38:1142–53. doi: 10.1016/j.immuni.2013.05.016 PMC3730833 23809161

[56] Verhoef PA Kertesy SB Lundberg K Kahlenberg JM Dubyak GR. Inhibitory Effects of Chloride on the Activation of Caspase-1, IL-1β Secretion, and Cytolysis by the P2X7 Receptor. J Immunol (2005) 175:7623–34. doi: 10.4049/jimmunol.175.11.7623 16301672

[57] Compan V Baroja-Mazo A López-Castejón G Gomez AI Martínez CM Angosto D. Cell Volume Regulation Modulates Nlrp3 Inflammasome Activation. Immunity (2012) 37:487–500. doi: 10.1016/j.immuni.2012.06.013 22981536

[58] Tang T Lang X Xu C Wang X Gong T Yang Y. Clics-Dependent Chloride Efflux Is an Essential and Proximal Upstream Event for NLRP3 Inflammasome Activation. Nat Commun (2017) 8:202. doi: 10.1038/s41467-017-00227-x 28779175 PMC5544706

[59] Lee G-S Subramanian N Kim AI Aksentijevich I Goldbach-Mansky R Sacks DB. The Calcium-Sensing Receptor Regulates the Nlrp3 Inflammasome Through Ca2+ and Camp. Nature (2012) 492:123–7. doi: 10.1038/nature11588 PMC4175565 23143333

[60] Murakami T Ockinger J Yu J Byles V McColl A Hofer AM. Critical Role for Calcium Mobilization in Activation of the NLRP3 Inflammasome. Proc Natl Acad Sci (2012) 109:11282–7. doi: 10.1073/pnas.1117765109 PMC3396518 22733741

[61] Rossol M Pierer M Raulien N Quandt D Meusch U Rothe K. Extracellular Ca2+ is a Danger Signal Activating the NLRP3 Inflammasome Through G Protein-Coupled Calcium Sensing Receptors. Nat Commun (2012) 3:1329. doi: 10.1038/ncomms2339 23271661 PMC3535422

[62] Zhou R Yazdi AS Menu P Tschopp J. A Role for Mitochondria in NLRP3 Inflammasome Activation. Nature (2011) 469:221–5. doi: 10.1038/nature09663 21124315

[63] Shimada K Crother TR Karlin J Dagvadorj J Chiba N Chen S. Oxidized Mitochondrial DNA Activates the NLRP3 Inflammasome During Apoptosis. Immunity (2012) 36:401–14. doi: 10.1016/j.immuni.2012.01.009 PMC3312986 22342844

[64] Groslambert M Py BF. Spotlight on the NLRP3 Inflammasome Pathway. J Inflammation Res (2018) 11:359–74. doi: 10.2147/jir.s141220 PMC6161739 30288079

[65] Yang J Zhao Y Shao F. Non-Canonical Activation of Inflammatory Caspases by Cytosolic LPS in Innate Immunity. Curr Opin Immunol (2015) 32:78–83. doi: 10.1016/j.coi.2015.01.007 25621708

[66] Kayagaki N Warming S Lamkanfi M Walle LV Louie S Dong J. Non-Canonical Inflammasome Activation Targets Caspase-11. Nature (2011) 479:117–21. doi: 10.1038/nature10558 22002608

[67] Broz P Ruby T Belhocine K Bouley DM Kayagaki N Dixit VM. Caspase-11 Increases Susceptibility to Salmonella Infection in the Absence of Caspase-1. Nature (2012) 490:288–91. doi: 10.1038/nature11419 PMC3470772 22895188

[68] Rathinam VAK Vanaja SK Waggoner L Sokolovska A Becker C Stuart LM. TRIF Licenses Caspase-11-Dependent NLRP3 Inflammasome Activation by Gram-Negative Bacteria. Cell (2012) 150:606–19. doi: 10.1016/j.cell.2012.07.007 PMC3660860 22819539

[69] Hagar JA Powell DA Aachoui Y Ernst RK Miao EA. Cytoplasmic LPS Activates Caspase-11: Implications in TLR4-Independent Endotoxic Shock. Science (2013) 341:1250–3. doi: 10.1126/science.1240988 PMC3931427 24031018

[70] Kayagaki N Wong MT Stowe IB Ramani SR Gonzalez LC Akashi-Takamura S. Noncanonical Inflammasome Activation by Intracellular LPS Independent of TLR4. Science (2013) 341:1246–9. doi: 10.1126/science.1240248 23887873

[71] Shi J Zhao Y Wang Y Gao W Ding J Li P. Inflammatory Caspases are Innate Immune Receptors for Intracellular LPS. Nature (2014) 514:187–92. doi: 10.1038/nature13683 25119034

[72] Shenoy AR Wellington DA Kumar P Kassa H Booth CJ Cresswell P. GBP5 Promotes NLRP3 Inflammasome Assembly and Immunity in Mammals. Science (2012) 336:481–5. doi: 10.1126/science.1217141 22461501

[73] Meunier E Dick MS Dreier RF Schürmann N Broz DK Warming S. Caspase-11 Activation Requires Lysis of Pathogen-Containing Vacuoles by IFN-Induced Gtpases. Nature (2014) 509:366–70. doi: 10.1038/nature13157 24739961

[74] Aachoui Y Leaf IA Hagar JA Fontana MF Campos CG Zak DE. Caspase-11 Protects Against Bacteria That Escape the Vacuole. Science (2013) 339:975–8. doi: 10.1126/science.1230751 PMC3697099 23348507

[75] Man SM Karki R Sasai M Place DE Kesavardhana S Temirov J. IRGB10 Liberates Bacterial Ligands for Sensing by the AIM2 and Caspase-11-NLRP3 Inflammasomes. Cell (2016) 167:382–396.e17. doi: 10.1016/j.cell.2016.09.012 27693356 PMC5074697

[76] Kutsch M Sistemich L Lesser CF Goldberg MB Herrmann C Coers J. Direct Binding of Polymeric GBP1 to LPS Disrupts Bacterial Cell Envelope Functions. EMBO J (2020) 39:e104926. doi: 10.15252/embj.2020104926 32510692 PMC7327485

[77] Santos JC Boucher D Schneider LK Demarco B Dilucca M Shkarina K. Human GBP1 Binds LPS to Initiate Assembly of a Caspase-4 Activating Platform on Cytosolic Bacteria. Nat Commun (2020) 11:3276. doi: 10.1038/s41467-020-16889-z 32581219 PMC7314798

[78] Wandel MP Kim B-H Park E-S Boyle KB Nayak K Lagrange B. Guanylate-Binding Proteins Convert Cytosolic Bacteria Into Caspase-4 Signaling Platforms. Nat Immunol (2020) 21:880–91. doi: 10.1038/s41590-020-0697-2 PMC7381384 32541830

[79] Yi Y. Functional Crosstalk Between non-Canonical Caspase-11 and Canonical NLRP3 Inflammasomes During Infection-Mediated Inflammation. Immunology (2020) 159:142–55. doi: 10.1111/imm.13134 PMC6954705 31630388

[80] Bürckstümmer T Baumann C Blüml S Dixit E Dürnberger G Jahn H. An Orthogonal Proteomic-Genomic Screen Identifies AIM2 as a Cytoplasmic DNA Sensor for the Inflammasome. Nat Immunol (2009) 10:266–72. doi: 10.1038/ni.1702 19158679

[81] Fernandes-Alnemri T Yu J-W Wu J Datta P Alnemri ES. AIM2 Activates the Inflammasome and Cell Death in Response to Cytoplasmic DNA. Nature (2009) 458:509–13. doi: 10.1038/nature07710 PMC2862225 19158676

[82] Hornung V Ablasser A Charrel-Dennis M Bauernfeind F Horvath G Caffrey DR. AIM2 Recognizes Cytosolic Dsdna and Forms a Caspase-1 Activating Inflammasome With ASC. Nature (2009) 458:514–8. doi: 10.1038/nature07725 PMC2726264 19158675

[83] Wang L-Q Liu T Yang S Sun L Zhao Z-Y Li L-Y. Perfluoroalkyl Substance Pollutants Activate the Innate Immune System Through the AIM2 Inflammasome. Nat Commun (2021) 12:2915. doi: 10.1038/s41467-021-23201-0 34006824 PMC8131593

[84] Moretti J Blander JM. Increasing Complexity of NLRP3 Inflammasome Regulation. J Leukocyte Biol (2021) 109:561–71. doi: 10.1002/jlb.3mr0520-104rr PMC8985609 32531835

[85] Netea MG Simon A van de Veerdonk F Kullberg B-J der Meer JWMV Joosten LAB. IL-1β Processing in Host Defense: Beyond the Inflammasomes. PloS Pathog (2010) 6:e1000661. doi: 10.1371/journal.ppat.1000661 20195505 PMC2829053

[86] Netea MG van de Veerdonk FL van der Meer JWM Dinarello CA Joosten LAB. Inflammasome-Independent Regulation of IL-1-Family Cytokines. Annu Rev Immunol (2014) 33:1–29. doi: 10.1146/annurev-immunol-032414-112306 25493334

[87] Coeshott C Ohnemus C Pilyavskaya A Ross S Wieczorek M Kroona H. Converting Enzyme-Independent Release of Tumor Necrosis Factor A and IL-1β From a Stimulated Human Monocytic Cell Line in the Presence of Activated Neutrophils or Purified Proteinase 3. Proc Natl Acad Sci (1999) 96:6261–6. doi: 10.1073/pnas.96.11.6261 PMC26869 10339575

[88] Sugawara S Uehara A Nochi T Yamaguchi T Ueda H Sugiyama A. Neutrophil Proteinase 3-Mediated Induction of Bioactive IL-18 Secretion by Human Oral Epithelial Cells. J Immunol (2001) 167:6568–75. doi: 10.4049/jimmunol.167.11.6568 11714826

[89] Herzog C Haun RS Kaushal V Mayeux PR Shah SV Kaushal GP. Meprin a and Meprin A Generate Biologically Functional IL-1β From Pro-IL-1β. Biochem Bioph Res Co (2009) 379:904–8. doi: 10.1016/j.bbrc.2008.12.161 PMC3702385 19135030

[90] Schönbeck U Mach F Libby P. Generation of Biologically Active IL-1 Beta by Matrix Metalloproteinases: A Novel Caspase-1-Independent Pathway of IL-1 Beta Processing. J Immunol Baltim Md 1950 (1998) 161:3340–6. 9759850

[91] Hildebrand D Bode KA Rieß D Cerny D Waldhuber A Römmler F. Granzyme a Produces Bioactive IL-1β Through a Nonapoptotic Inflammasome-Independent Pathway. Cell Rep (2014) 9:910–7. doi: 10.1016/j.celrep.2014.10.003 25437548

[92] Orning P Lien E. Multiple Roles of Caspase-8 in Cell Death, Inflammation, and Innate Immunity. J Leukocyte Biol (2021) 109:121–41. doi: 10.1002/jlb.3mr0420-305r PMC8664275 32531842

[93] Maelfait J Vercammen E Janssens S Schotte P Haegman M Magez S. Stimulation of Toll-Like Receptor 3 and 4 Induces Interleukin-1β Maturation by Caspase-8. J Exp Med (2008) 205:1967–73. doi: 10.1084/jem.20071632 PMC2526192 18725521

[94] Antonopoulos C Sanadi CE Kaiser WJ Mocarski ES Dubyak GR. Proapoptotic Chemotherapeutic Drugs Induce Noncanonical Processing and Release of IL-1β *via* Caspase-8 in Dendritic Cells. J Immunol (2013) 191:4789–803. doi: 10.4049/jimmunol.1300645 PMC3870469 24078693

[95] Shenderov K Riteau N Yip R Mayer-Barber KD Oland S Hieny S. Cutting Edge: Endoplasmic Reticulum Stress Licenses Macrophages to Produce Mature IL-1β in Response to TLR4 Stimulation Through a Caspase-8– and TRIF-Dependent Pathway. J Immunol (2014) 192:2029–33. doi: 10.4049/jimmunol.1302549 PMC3935725 24489101

[96] Holler N Zaru R Micheau O Thome M Attinger A Valitutti S. Fas Triggers an Alternative, Caspase-8–Independent Cell Death Pathway Using the Kinase RIP as Effector Molecule. Nat Immunol (2000) 1:489–95. doi: 10.1038/82732 11101870

[97] Bossaller L Chiang P-I Schmidt-Lauber C Ganesan S Kaiser WJ Rathinam VAK. Cutting Edge: FAS (CD95) Mediates Noncanonical IL-1β and IL-18 Maturation via Caspase-8 in an RIP3-Independent Manner. J Immunol (2012) 189:5508–12. doi: 10.4049/jimmunol.1202121 PMC3518757 23144495

[98] Vince JE Wong WW-L Gentle I Lawlor KE Allam R O’Reilly L. Inhibitor of Apoptosis Proteins Limit RIP3 Kinase-Dependent Interleukin-1 Activation. Immunity (2012) 36:215–27. doi: 10.1016/j.immuni.2012.01.012 22365665

[99] Moriwaki K Bertin J Gough PJ Chan FK-M. A RIPK3–Caspase 8 Complex Mediates Atypical Pro–IL-1β Processing. J Immunol (2015) 194:1938–44. doi: 10.4049/jimmunol.1402167 PMC4324020 25567679

[100] Briard B Malireddi RKS Kanneganti T-D. Role of Inflammasomes/Pyroptosis and Panoptosis During Fungal Infection. PloS Pathog (2021) 17:e1009358. doi: 10.1371/journal.ppat.1009358 33735255 PMC7971547

[101] Gringhuis SI Kaptein TM Wevers BA Theelen B van der Vlist M Boekhout T. Dectin-1 is an Extracellular Pathogen Sensor for the Induction and Processing of IL-1β via a Noncanonical Caspase-8 Inflammasome. Nat Immunol (2012) 13:246–54. doi: 10.1038/ni.2222 22267217

[102] Mayer-Barber KD Sher A. Cytokine and Lipid Mediator Networks in Tuberculosis. Immunol Rev (2015) 264:264–75. doi: 10.1111/imr.12249 PMC4339232 25703565

[103] Orme IM Robinson RT Cooper AM. The Balance Between Protective and Pathogenic Immune Responses in the TB-Infected Lung. Nat Immunol (2015) 16:57–63. doi: 10.1038/ni.3048 25521685

[104] Domingo-Gonzalez R Prince O Cooper A Khader SA. Cytokines and Chemokines in Mycobacterium Tuberculosis Infection. Microbiol Spectr (2016) 4(5):10.1128/microbiolspec.TBTB2-0018-2016. doi: 10.1128/microbiolspec.tbtb2-0018-2016 PMC5205539 27763255

[105] Simmons JD Stein CM Seshadri C Campo M Alter G Fortune S. Immunological Mechanisms of Human Resistance to Persistent Mycobacterium Tuberculosis Infection. Nat Rev Immunol (2018) 18:575–89. doi: 10.1038/s41577-018-0025-3 PMC6278832 29895826

[106] Sia JK Rengarajan J. Immunology of Mycobacterium Tuberculosis Infections. Microbiol Spectr (2019) 7(4):10.1128/microbiolspec.GPP3-0022-2018. doi: 10.1128/microbiolspec.gpp3-0022-2018 PMC6636855 31298204

[107] Juffermans NP Florquin S Camoglio L Verbon A Kolk AH Speelman P. Interleukin-1 Signaling is Essential for Host Defense During Murine Pulmonary Tuberculosis. J Infect Dis (2000) 182:902–8. doi: 10.1086/315771 10950787

[108] Yamada H Mizumo S Horai R Iwakura Y Sugawara I. Protective Role of Interleukin-1 in Mycobacterial Infection in IL-1 Alpha/Beta Double-Knockout Mice. Lab Invest (2000) 80:759–67. doi: 10.1038/labinvest.3780079 10830786

[109] Sugawara I Yamada H Hua S Mizuno S. Role of Interleukin (IL)-1 Type 1 Receptor in Mycobacterial Infection. Microbiol Immunol (2001) 45:743–50. doi: 10.1111/j.1348-0421.2001.tb01310.x 11791667

[110] Fremond CM Togbe D Doz E Rose S Vasseur V Maillet I. IL-1 Receptor-Mediated Signal is an Essential Component of Myd88-Dependent Innate Response to Mycobacterium Tuberculosis Infection. J Immunol (2007) 179:1178–89. doi: 10.4049/jimmunol.179.2.1178 17617611

[111] Mayer-Barber KD Barber DL Shenderov K White SD Wilson MS Cheever A. Caspase-1 Independent IL-1beta Production is Critical for Host Resistance to Mycobacterium Tuberculosis and Does Not Require TLR Signaling in Vivo. J Immunol (Baltimore Md: 1950) (2010) 184:3326–30. doi: 10.4049/jimmunol.0904189 PMC3420351 20200276

[112] Mayer-Barber KD Andrade BB Barber DL Hieny S Feng CG Caspar P. Innate and Adaptive Interferons Suppress IL-1α and IL-1β Production by Distinct Pulmonary Myeloid Subsets During Mycobacterium Tuberculosis Infection. Immunity (2011) 35:1023–34. doi: 10.1016/j.immuni.2011.12.002 PMC3246221 22195750

[113] Jayaraman P Sada-Ovalle I Nishimura T Anderson AC Kuchroo VK Remold HG. IL-1β Promotes Antimicrobial Immunity in Macrophages by Regulating TNFR Signaling and Caspase-3 Activation. J Immunol (Baltimore Md. : 1950) (2013) 190:4196–204. doi: 10.4049/jimmunol.1202688 PMC3622150 23487424

[114] Pilli M Arko-Mensah J Ponpuak M Roberts E Master S Mandell MA. TBK-1 Promotes Autophagy-Mediated Antimicrobial Defense by Controlling Autophagosome Maturation. Immunity (2012) 37:223–34. doi: 10.1016/j.immuni.2012.04.015 PMC3428731 22921120

[115] Master SS Rampini SK Davis AS Keller C Ehlers S Springer B. Mycobacterium Tuberculosis Prevents Inflammasome Activation. Cell Host Microbe (2008) 3:224–32. doi: 10.1016/j.chom.2008.03.003 PMC3657562 18407066

[116] Eklund D Welin A Andersson H Verma D Söderkvist P Stendahl O. Human Gene Variants Linked to Enhanced NLRP3 Activity Limit Intramacrophage Growth of Mycobacterium Tuberculosis. J Infect Dis (2014) 209:749–53. doi: 10.1093/infdis/jit572 PMC3923544 24158955

[117] Bohrer AC Tocheny C Assmann M Ganusov VV Mayer-Barber KD. Cutting Edge: IL-1R1 Mediates Host Resistance to Mycobacterium Tuberculosis by Trans-Protection of Infected Cells. J Immunol (Baltimore Md. : 1950) (2018) 201:1645–50. doi: 10.4049/jimmunol.1800438 PMC6125209 30068597

[118] Mayer-Barber KD Andrade BB Oland SD Amaral EP Barber DL Gonzales J. Host-Directed Therapy of Tuberculosis Based on Interleukin-1 and Type I Interferon Crosstalk. Nature (2014) 511:99–103. doi: 10.1038/nature13489 24990750 PMC4809146

[119] Dorhoi A Kaufmann SHE. Pathology and Immune Reactivity: Understanding Multidimensionality in Pulmonary Tuberculosis. Semin Immunopathol (2016) 38:153–66. doi: 10.1007/s00281-015-0531-3 26438324

[120] Moreira-Teixeira L Mayer-Barber K Sher A O’Garra A. Type I Interferons in Tuberculosis: Foe and Occasionally Friend. J Exp Med (2018) 215:1273–85. doi: 10.1084/jem.20180325 PMC5940272 29666166

[121] Mayer-Barber KD Yan B. Clash of the Cytokine Titans: Counter-Regulation of Interleukin-1 and Type I Interferon-Mediated Inflammatory Responses. Cell Mol Immunol (2017) 14:22–35. doi: 10.1038/cmi.2016.25 27264686 PMC5214938

[122] Zhang L Jiang X Pfau D Ling Y Nathan CF. Type I Interferon Signaling Mediates Mycobacterium Tuberculosis–Induced Macrophage Death. J Exp Med (2020) 218:556–16. doi: 10.1084/jem.20200887 PMC7608065 33125053

[123] Sousa J Cá B Maceiras AR Simões-Costa L Fonseca KL Fernandes AI. Mycobacterium Tuberculosis Associated With Severe Tuberculosis Evades Cytosolic Surveillance Systems and Modulates IL-1β Production. Nat Commun (2020) 11:1949. doi: 10.1038/s41467-020-15832-6 32327653 PMC7181847

[124] Matty MA Knudsen DR Walton EM Beerman RW Cronan MR Pyle CJ. Potentiation of P2RX7 as a Host-Directed Strategy for Control of Mycobacterial Infection. Elife (2019) 8:e39123. doi: 10.7554/elife.39123 30693866 PMC6351102

[125] Zhang G Zhou B Li S Yue J Yang H Wen Y. Allele-Specific Induction of IL-1β Expression by C/Ebpβ and PU.1 Contributes to Increased Tuberculosis Susceptibility. PloS Pathog (2014) 10:e1004426. doi: 10.1371/journal.ppat.1004426 25329476 PMC4199770

[126] Wilkinson RJ Patel P Llewelyn M Hirsch CS Pasvol G Snounou G. Influence of Polymorphism in the Genes for the Interleukin (IL)-1 Receptor Antagonist and IL-1β on Tuberculosis. J Exp Med (1999) 189:1863–74. doi: 10.1084/jem.189.12.1863 PMC2192963 10377182

[127] Mishra BB Rathinam VAK Martens GW Martinot AJ Kornfeld H Fitzgerald KA. Nitric Oxide Controls the Immunopathology of Tuberculosis by Inhibiting NLRP3 Inflammasome–Dependent Processing of IL-1β. Nat Immunol (2012) 14:52–60. doi: 10.1038/ni.2474 23160153 PMC3721324

[128] Mishra BB Lovewell RR Olive AJ Zhang G Wang W Eugenin E. Nitric Oxide Prevents a Pathogen-Permissive Granulocytic Inflammation During Tuberculosis. Nat Microbiol (2017) 2:17072. doi: 10.1038/nmicrobiol.2017.72 28504669 PMC5461879

[129] Subbarao S Sanchez-Garrido J Krishnan N Shenoy AR Robertson BD. Genetic and Pharmacological Inhibition of Inflammasomes Reduces the Survival of Mycobacterium Tuberculosis Strains in Macrophages. Sci Rep-uk (2020) 10:3709. doi: 10.1038/s41598-020-60560-y PMC7048741 32111888

[130] Roca FJ Ramakrishnan L. TNF Dually Mediates Resistance and Susceptibility to Mycobacteria via Mitochondrial Reactive Oxygen Species. Cell (2013) 153:521–34. doi: 10.1016/j.cell.2013.03.022 PMC3790588 23582643

[131] Taft J Bogunovic D. The Goldilocks Zone of Type I Ifns: Lessons From Human Genetics. J Immunol (Baltimore Md: 1950) (2018) 201:3479–85. doi: 10.4049/jimmunol.1800764 30530500

[132] Zheng D Liwinski T Elinav E. Inflammasome Activation and Regulation: Toward a Better Understanding of Complex Mechanisms. Cell Discov (2020) 6:36. doi: 10.1038/s41421-020-0167-x 32550001 PMC7280307

[133] TeKippe EM Allen IC Hulseberg PD Sullivan JT McCann JR Sandor M. Granuloma Formation and Host Defense in Chronic Mycobacterium Tuberculosis Infection Requires PYCARD/ASC But Not NLRP3 or Caspase-1. PloS One (2010) 5:e12320. doi: 10.1371/journal.pone.0012320 20808838 PMC2924896

[134] Rastogi S Ellinwood S Augenstreich J Mayer-Barber KD Briken V. Mycobacterium Tuberculosis Inhibits the NLRP3 Inflammasome Activation via its Phosphokinase Pknf. PloS Pathog (2021) 17:e1009712. doi: 10.1371/journal.ppat.1009712 34324582 PMC8321130

[135] Beckwith KS Beckwith MS Ullmann S Sætra RS Kim H Marstad A. Plasma Membrane Damage Causes NLRP3 Activation and Pyroptosis During Mycobacterium Tuberculosis Infection. Nat Commun (2020) 11:2270. doi: 10.1038/s41467-020-16143-6 32385301 PMC7210277

[136] Mendes ALG Joaquim HDM Zamae MIS Assis RM Peixoto JR de M. Expression of NLRP3 Inflammasome in Leprosy Indicates Immune Evasion of Mycobacterium Leprae. Memórias Inst Oswaldo Cruz (2020) 115:e190324. doi: 10.1590/0074-02760190324 PMC7046136 32130367

[137] Ligon LS Hayden JD Braunstein M. The Ins and Outs of Mycobacterium Tuberculosis Protein Export. Tuberc (Edinburgh Scotland) (2012) 92:121–32. doi: 10.1016/j.tube.2011.11.005 PMC3288827 22192870

[138] Majlessi L Rosales RP Casadevall A Brosch R. Release of Mycobacterial Antigens. Immunol Rev (2015) 264:25–45. doi: 10.1111/imr.12251 25703550

[139] Vaziri F Brosch R. ESX/Type VII Secretion Systems—An Important Way Out for Mycobacterial Proteins. Microbiol Spectr (2019) 7:351–62. doi: 10.1128/microbiolspec.psib-0029-2019 PMC10957191 31298207

[140] Augenstreich J Briken V. Host Cell Targets of Released Lipid and Secreted Protein Effectors of Mycobacterium Tuberculosis. Front Cell Infect Microbiol (2020) 10:595029. doi: 10.3389/fcimb.2020.595029 33194845 PMC7644814

[141] Pathak SK Basu S Basu KK Banerjee A Pathak S Bhattacharyya A. Direct Extracellular Interaction Between the Early Secreted Antigen ESAT-6 of Mycobacterium Tuberculosis and TLR2 Inhibits TLR Signaling in Macrophages. Nat Immunol (2007) 8:610–8. doi: 10.1038/ni1468 17486091

[142] Chatterjee S Dwivedi VP Singh Y Siddiqui I Sharma P Kaer LV. Early Secreted Antigen ESAT-6 of Mycobacterium Tuberculosis Promotes Protective T Helper 17 Cell Responses in a Toll-Like Receptor-2-Dependent Manner. PloS Pathog (2011) 7:e1002378. doi: 10.1371/journal.ppat.1002378 22102818 PMC3213116

[143] Kumar P Agarwal R Siddiqui I Vora H Das G Sharma P. ESAT6 Differentially Inhibits IFN-Γ-Inducible Class II Transactivator Isoforms in Both a TLR2-Dependent and -Independent Manner. Immunol Cell Biol (2012) 90:411–20. doi: 10.1038/icb.2011.54 21670739

[144] Jang A-R Choi J-H Shin SJ Park J-H. Mycobacterium Tuberculosis ESAT6 Induces IFN-B Gene Expression in Macrophages via Tlrs-Mediated Signaling. Cytokine (2017) 104:104–9. doi: 10.1016/j.cyto.2017.10.006 29046251

[145] Schweneker K Gorka O Schweneker M Poeck H Tschopp J Peschel C. The Mycobacterial Cord Factor Adjuvant Analogue Trehalose-6,6′-Dibehenate (TDB) Activates the Nlrp3 Inflammasome. Immunobiology (2013) 218:664–73. doi: 10.1016/j.imbio.2012.07.029 22921586

[146] Manzanillo PS Shiloh MU Portnoy DA Cox JS. Mycobacterium Tuberculosis Activates the DNA-Dependent Cytosolic Surveillance Pathway Within Macrophages. Cell Host Microbe (2012) 11:469–80. doi: 10.1016/j.chom.2012.03.007 PMC3662372 22607800

[147] Sauer J-D Witte CE Zemansky J Hanson B Lauer P Portnoy DA. Listeria Monocytogenes Triggers AIM2-Mediated Pyroptosis Upon Infrequent Bacteriolysis in the Macrophage Cytosol. Cell Host Microbe (2010) 7:412–9. doi: 10.1016/j.chom.2010.04.004 PMC2947455 20417169

[148] Madan-Lala R Peixoto KV Re F Rengarajan J. Mycobacterium Tuberculosis Hip1 Dampens Macrophage Proinflammatory Responses by Limiting Toll-Like Receptor 2 Activation. Infect Immun (2011) 79:4828–38. doi: 10.1128/iai.05574-11 PMC3232659 21947769

[149] Naffin-Olivos JL Georgieva M Goldfarb N Madan-Lala R Dong L Bizzell E. Mycobacterium Tuberculosis Hip1 Modulates Macrophage Responses Through Proteolysis of Groel2. PloS Pathog (2014) 10:e1004132. doi: 10.1371/journal.ppat.1004132 24830429 PMC4022732

[150] Danelishvili L Everman JL McNamara MJ Bermudez LE. Inhibition of the Plasma-Membrane-Associated Serine Protease Cathepsin G by Mycobacterium Tuberculosis Rv3364c Suppresses Caspase-1 and Pyroptosis in Macrophages. Front Microbiol (2012) 2:281. doi: 10.3389/fmicb.2011.00281 22275911 PMC3257866

[151] Hernandez-Cuellar E Tsuchiya K Hara H Fang R Sakai S Kawamura I. Cutting Edge: Nitric Oxide Inhibits the NLRP3 Inflammasome. J Immunol (2012) 189:5113–7. doi: 10.4049/jimmunol.1202479 23100513

[152] Hortle E Tran LV Fontaine AR Pinello N Wong JJ-L Britton WJ. OXSR1 Inhibits Inflammasome Activation by Limiting Potassium Efflux During Mycobacterial Infection. Biorxiv (2021) 2021.04.21.440692. doi: 10.1101/2021.04.21.440692 PMC9107790 35545295

[153] Zhang Q Jiang X He W Wei K Sun J Qin X. MCL Plays an Anti-Inflammatory Role in Mycobacterium Tuberculosis-Induced Immune Response by Inhibiting NF-Kb and NLRP3 Inflammasome Activation. Mediat Inflammation (2017) 2017:1–12. doi: 10.1155/2017/2432904 PMC5470027 28642632

[154] Pelegrin P Barroso-Gutierrez C Surprenant A. P2X7 Receptor Differentially Couples to Distinct Release Pathways for IL-1β in Mouse Macrophage. J Immunol (2008) 180:7147–57. doi: 10.4049/jimmunol.180.11.7147 18490713

[155] Briken V Ahlbrand SE Shah S. Mycobacterium Tuberculosis and the Host Cell Inflammasome: A Complex Relationship. Front Cell Infect Mi (2013) 3:62. doi: 10.3389/fcimb.2013.00062 PMC3793174 24130966

